# Association of Medicaid Expansion With Health Insurance Coverage Among Persons With a Disability

**DOI:** 10.1001/jamanetworkopen.2019.7136

**Published:** 2019-07-17

**Authors:** Jim P. Stimpson, Jessie Kemmick Pintor, Ryan M. McKenna, Sungchul Park, Fernando A. Wilson

**Affiliations:** 1 Dornsife School of Public Health, Drexel University, Philadelphia, Pennsylvania; 2 University of Nebraska Medical Center, College of Public Health, Omaha

## Abstract

**Importance:**

Although nearly 1 in 5 persons in the United States has a physical or mental disability, little is known about the association of the Patient Protection and Affordable Care Act (ACA) with health insurance coverage among persons with a disability.

**Objective:**

To determine the association of Medicaid expansion with health insurance coverage among persons with a disability.

**Design, Setting, and Participants:**

Cross-sectional analysis of adults eligible for Medicaid expansion (aged 26-64 years with incomes up to 138% of the federal poverty level), using a triple-differences (difference-in-difference-in-difference) approach to compare the pre-ACA with post-ACA trend in health insurance rates by disability status between expansion and nonexpansion states using nationally representative, repeated cross-sectional sample data obtained from the American Community Survey in the United States from January 1, 2010, to December 31, 2016. Time was defined as either pre-ACA (January 1, 2010, to December 31, 2013) or post-ACA (January 1, 2014, to December 31, 2016). Treatment status was defined as whether a state implemented Medicaid expansion after January 1, 2014. States that expanded Medicaid between January 1, 2014, to December 31, 2016, were classified as the treatment group, and states that did not expand Medicaid during the study period were classified as the control group. Data were analyzed from December 12, 2018, to May 21, 2019.

**Main Outcomes and Measures:**

Self-reported health insurance coverage (uninsured, Medicaid, private) and self-reported disability status (≥1 condition limiting activity, including cognitive, ambulatory, self-care, independent living, and sensory difficulties).

**Results:**

Of 2 549 376 Medicaid-eligible adults, 1 348 620 (52.9%) were female; 1 218 602 (47.8%) were non-Hispanic white, 497 128 (19.5%) were non-Hispanic black, 211 598 (8.3%) were Hispanic, and 206 499 (8.1%) were of other race/ethnicity; and 619 498 (24.3%) reported at least 1 disability. The percentage of persons without health insurance was greatest for persons without a disability who lived in a nonexpansion state before the ACA’s Medicaid expansion provision went into effect (236 645 of 426 387 [55.5%]), and the smallest proportion of persons without health insurance was reported for persons with a disability living in an expansion state after the ACA went into effect (19 552 of 176 145 [11.1%]). Triple-differences analysis suggested that Medicaid expansion was associated with a decrease in the uninsured rate for both persons with a disability (7.1% − 16.2% = −9.1%) and without a disability (21.2% − 34.9% = −13.7%) and that Medicaid expansion was associated with a 4.6% decrease in the uninsurance rate for persons without a disability and a 2.6% decrease in persons with a disability (*P* < .001). Although Medicaid expansion was associated with an increase in Medicaid coverage for both persons with a disability (49.3% pre-ACA to 62.3% post-ACA; change, 13.0%) and persons without a disability (21.6% pre-ACA to 40.3% post-ACA; change, 17.7%), the triple difference–estimated Medicaid coverage was −4.7% for persons with a disability and 0.4% for persons without a disability, a difference of 5.1% (*P* < .001). Medicaid expansion was associated with a 3% higher private insurance rate for persons with a disability than for persons without a disability.

**Conclusions and Relevance:**

Medicaid expansion appeared to be associated with lower uninsurance rates and higher Medicaid and private insurance coverage for persons with a disability. This study’s findings suggest that the reduction in the uninsured rate and gains in Medicaid coverage were greater for persons without a disability than for persons with a disability.

## Key Points

**Question:**

Was the Patient Protection and Affordable Care Act Medicaid expansion associated with an increase in health insurance coverage among persons with a disability?

**Findings:**

In this cross-sectional analysis of more than 2.5 million US adults aged 26 to 64 years with incomes up to 138% of the federal poverty level, Medicaid expansion was significantly associated with lower uninsurance rates and higher Medicaid and private insurance coverage for persons with a disability.

**Meaning:**

These findings suggest that Medicaid expansion was associated with improved health insurance coverage among persons with a disability.

## Teaser

This cross-sectional analysis of data from more than 2.5 million Medicaid-eligible US adults in states that enacted and did not enact Medicaid expansion under the Patient Protection and Affordable Care Act evaluates the association of Medicaid expansion with health insurance coverage among persons with a disability.

## Introduction

Medicaid expansion was a provision of the Patient Protection and Affordable Care Act (ACA) that was intended to increase access to Medicaid coverage for adults with incomes up to 138% of the federal poverty level.^1^ A Supreme Court decision in June 2012, *National Federation of Independent Business v Sebelius*, made Medicaid expansion optional for states, rather than mandatory as the ACA had originally intended. Consequently, 25 states expanded Medicaid eligibility by January 1, 2014, the implementation date set by the ACA, and 9 states expanded after that date. The results of the 2018 midterm elections will add 3 more states to the Medicaid expansion list, and other states that had a change in governorship may follow suit.

There has been a burgeoning evidence base on the association of Medicaid expansion since 2014 with various outcomes.^2,3^ Overall, expansion of Medicaid has not only reduced the number of uninsured persons but has also improved access to care, use, affordability, and health outcomes.^4,5,6,7,8,9,10,11,12^ Medicaid expansion has been positively associated with greater revenue in state budgets, lower state unemployment, and improved financial outcomes for hospitals, especially rural hospitals.^13,14,15,16,17,18^ Moreover, certain populations in need of health care access, including persons with chronic diseases, mental health conditions, and members of racial/ethnic minority groups and immigrants, have seen improvement in access and in outcomes.^19,20,21,22,23^

However, we have limited information on the association of the ACA Medicaid expansion with coverage and outcomes among persons with a disability, although nearly 1 in 5 persons in the United States has a physical or mental disability.^24^ Persons with a disability have higher rates of Medicaid coverage and lower uninsurance rates than persons without a disability. However, before the ACA, access to health insurance for persons with a disability was complicated for a variety of reasons, including lower likelihood of employer-based coverage, reduced access to private insurance owing to preexisting conditions, and income restrictions for Medicaid coverage that are below the federal poverty level across the country.^25,26^ Evidence from a recent nationally representative trend study found improvements for persons with a disability in insurance coverage, access, use, and affordability after 2014.^26^ The ACA Medicaid expansion made it easier for persons with a disability to receive Medicaid coverage because the income threshold was raised to 138% of the federal poverty level and because it is not necessary to prove disability status to gain benefits. However, the specific association of Medicaid expansion with health insurance coverage for persons with a disability is still unknown.

In this study, we linked data on whether and when a state adopted Medicaid expansion with repeated cross-sectional data on health insurance coverage from the American Community Survey (ACS). We used a quasi-experimental approach to identify differences in health insurance coverage for persons with a disability by implementation of Medicaid expansion in their state of residence. Based on findings of other studies of the ACA^19,20,21,22,23^ and because this population tends to have greater need for health care and the ACA reduced barriers for adults to qualify for Medicaid coverage, we hypothesized that persons with a disability living in Medicaid expansion states would show greater improvement in health insurance coverage compared with persons with a disability living in states that chose not to expand Medicaid.

## Methods

### Data

This cross-sectional study followed the Strengthening the Reporting of Observational Studies in Epidemiology (STROBE) reporting guideline.^27^

We used the 1-year estimates from ACS data provided by the Integrated Public Use Microdata Series.^28^ The date range for the ACS data we used was January 1, 2010, to December 31, 2016. The ACS is a repeated cross-sectional survey conducted by the US Census Bureau to collect social and economic characteristics of persons and households and includes the Federal Information Processing Standards code, which permit the data to be linked with other databases at the state and county levels. The smallest identifiable geographic unit is the Public Use Microdata Area, containing at least 100 000 persons. Public Use Microdata Areas do not cross state boundaries. The ACS identifies the person who provided most or all of the information about the household to the enumerator. Because we used publicly available secondary data, the University of Nebraska Medical Center institutional review board deemed this study exempt from institutional review board review under federal category 4 and waived informed consent.

Data about state decisions on Medicaid expansion, including the date of implementation, came from the Henry J. Kaiser Family Foundation’s State Health Facts database.^29^ We added the state Federal Information Processing Standards codes to the Kaiser database and then linked it to the ACS data using the state identifier. The combined databases enabled the analysis of individual outcomes for persons living in a state that implemented Medicaid expansion.

Persons included in the study were adults aged 26 to 64 years, because the ACA allows persons up to 25 years of age to obtain coverage through their parents’ private health insurance provider, and had incomes at or below 138% of the federal poverty level. We determined poverty levels using the health insurance unit method.^30^ The data provided by the Integrated Public Use Microdata Series are complete with no missing data.^31^ The final analytic sample size was 2 549 376 respondents. Pooled characteristics of the sample can be found in eTable 1 in the Supplement. Time was defined as either pre-ACA (January 1, 2010, to December 31, 2013) or post-ACA (January 1, 2014, to December 31, 2016). Data were analyzed from December 12, 2018, to May 21, 2019.

### Outcome Measures

The outcome variable was whether the respondent had any health insurance coverage, Medicaid coverage, or private health insurance coverage. The predictor variable was disability, defined in the ACS as a reporting of 1 or more conditions limiting activity that included cognitive, ambulatory, self-care, independent living, and sensory difficulties.^32^ The Social Security Administration’s rules for defining disability require that a person have 1 or more medically diagnosed physical or mental impairments that are expected to prevent the person from substantial gainful activity for at least 12 months.^33^ An important difference between the Social Security Administration definition and our measure is that in ours, disability is self-reported by the respondent rather than medically determined. The trend in health insurance coverage by disability category can be found in the eFigure in the Supplement.

Control variables included sex (female or male), age (26-44 or 45-64 years), race/ethnicity (non-Hispanic white, Hispanic, non-Hispanic black, or other race/ethnicity), immigration status (born in the United States, naturalized citizen, noncitizen), marital status (married or unmarried), level of education (less than high school, high school, some college, or college), employment status (unemployed or employed), residence in a metropolitan area, and poverty status (income of 0% to <100% vs 100% to 138% of the federal poverty level).

### Statistical Analysis

We used a triple-differences (difference-in-difference-in-difference) approach to compare the pre-ACA and post-ACA trend in rate of health insurance coverage by disability status between expansion and nonexpansion states. This method isolates the change in health insurance coverage in the study period that was associated with Medicaid expansion compared with what the rate would have been if the state had not expanded Medicaid. Our triple-differences analysis measured the association of Medicaid expansion with rate of health insurance coverage through a 3-way interaction by population group (disability status), time (pre-ACA and post-ACA period), and treatment (state Medicaid expansion status). For ease of interpretation and to enable comparison with other ACA studies, we estimated linear probability regression models. Data shown in the eAppendix in the Supplement suggest that the parallel trends assumption for triple-differences analysis was met through the regression estimator and a visual inspection of the line graphs. We defined time as either pre-ACA (2010-2013) or post-ACA (2014-2016). The treatment effect was defined by whether a state implemented Medicaid expansion after January 1, 2014. States that expanded Medicaid between January 1, 2014, and December 31, 2016, were classified as the treatment group, and states that did not expand Medicaid during the study period were classified as the control group.

We conducted several sensitivity analyses, which are available in eTables 2 through 6 in the Supplement. We reanalyzed the triple-differences models for each health insurance outcome variable after excluding states that expanded Medicaid eligibility before January 1, 2014, or after December 31, 2014, referred to herein as *late expanders*. We also analyzed health insurance coverage through an employer or union, which is encapsulated in our private health insurance outcome variable. We excluded Medicare coverage from the triple-differences model for Medicaid coverage outcome to determine whether results were different when dual-eligible respondents were included in the population of persons with a disability. Finally, we estimated triple-differences models for each type of disability to ensure that the results were not being driven by the aggregated measure of disability status.

All analyses were conducted using Stata statistical software MP, version 15 MP (StataCorp) and accounted for survey weights and robust SEs. All triple difference models were analyzed using the *diff* command in Stata MP and adjusted for year and state fixed effects. Statistical significance was assumed at 2-sided *P* < .05.

## Results

Of 2 549 376 Medicaid-eligible adults aged 26 to 64 years analyzed, 1 348 620 (52.9%) were female; 1 218 602 (47.8%) were non-Hispanic white, 497 128 (19.5%) were non-Hispanic black, 211 598 (8.3%) were Hispanic, and 206 499 (8.1%) were of other race/ethnicity Among this sample, 619 498 (24.3%) reported at least 1 disability, 606 751 (23.8%) were born outside the United States, and 1 560 218 (61.2%) were not employed. More details about the sample characteristics can be found in eTable 1 in the Supplement. The weighted trend in insurance coverage for the study population by type of disability, found in the eFigure in the Supplement, indicated a downward trend in the uninsured population for each disability category with the steepest decline appearing in 2014. Likewise, there was an upward trend in coverage from Medicaid or private insurance. The pattern over time appears to be similar for each category of disability and insurance coverage type.

Table 1 shows the survey weighted characteristics for persons eligible for Medicaid before and after the ACA Medicaid expansion went into effect. A lower proportion of the study population reported being uninsured (29.49% post-ACA vs 42.96% pre-ACA), and a greater proportion reported having Medicaid (41.03% post-ACA vs 31.87% pre-ACA) or private insurance (28.69% post-ACA vs 23.91% pre-ACA) after the ACA went into effect. The proportion of the study population with a disability was less than 1% greater (0.7%) after the ACA went into effect (23.7% pre-ACA vs 24.4% post-ACA). There were no notable changes in the control variables over the study period.

**Table 1.** Weighted Characteristics of 2 549 376 Medicaid-Eligible Adults Before and After the ACA Medicaid Expansion, 2010-2016^a^

Variable	% (95% CI)	
	Pre-ACA, 2010-2013	Post-ACA, 2014-2016
Uninsured	43.0 (42.9-43.1)	29.5 (29.4-29.6)
Medicaid	31.8 (31.7-31.9)	41.0 (40.9-41.2)
Private insurance	23.9 (23.8-24.0)	28.7 (28.6-28.8)
Any disability	23.7 (23.6-23.8)	24.4 (24.3-24.5)
No. of disabilities		
0	76.3 (76.2-76.4)	75.6 (75.5-75.7)
1	8.0 (8.0-8.1)	8.2 (8.2-8.3)
2	15.6 (15.6-15.7)	16.2 (16.1-16.3)
Female sex	52.7 (52.6-52.8)	53.2 (53.1-53.3)
Aged 26-44 y	57.2 (57.1-57.3)	56.4 (56.3-56.5)
Aged 45-64 y	42.8 (42.7-42.9)	43.6 (43.5-43.7)
Income <100% FPL	73.7 (73.6-73.8)	73.2 (73.1-73.3)
Married	31.6 (31.5-31.7)	30.0 (30.0-30.1)
Race/ethnicity		
Non-Hispanic white	48.2 (48.1-48.3)	47.4 (47.2-47.5)
Hispanic	24.3 (24.2-24.4)	24.4 (24.3-24.6)
Non-Hispanic black	19.5 (19.4-19.6)	19.6 (19.5-19.7)
Other race/ethnicity	8.0 (8.0-8.1)	8.6 (8.5-8.6)
US native	76.1 (76.0-76.2)	76.4 (76.3-76.5)
Naturalized citizen	6.9 (6.9-7.0)	7.5 (7.4-7.6)
Noncitizen	17.0 (16.9-17.1)	16.1 (16.0-16.2)
Education level		
Less than high school	24.5 (24.4-24.6)	22.6 (22.5-22.7)
High school	43.6 (43.5-43.7)	44.0 (43.9-44.1)
Some college	21.1 (21.0-21.2)	21.6 (21.4-21.6)
College	10.9 (10.8-11.0)	11.8 (11.8-11.9)
Not employed	61.9 (61.8-62.0)	60.2 (60.0-60.3)
Metropolitan residence	75.7 (75.6-75.8)	76.4 (76.3-76.5)

Abbreviation: ACA, Patient Protection and Affordable Care Act; FPL, federal poverty level.

^a^ Medicaid-eligible defined as persons aged 26 to 64 years with income up to 138% of the FPL. Data are from the American Community Survey. All years are calendar years (January 1 through December 31).

Table 2 shows the weighted percentage of health insurance coverage for Medicaid-eligible persons stratified by time period, state Medicaid expansion status, and disability status. The percentage of persons without health insurance was greatest for persons without a disability who lived in a nonexpansion state before the ACA’s Medicaid expansion provision went into effect (236 645 of 426 387 [55.5%]; *P* < .001) and the smallest proportion of persons without health insurance was reported for persons with a disability living in an expansion state after the ACA went into effect (19 552 of 176 145 [11.1%];* P* < .001). Medicaid coverage was greatest for persons with disabilities living in an expansion state (123 654 of 176 145 [70.2%];* P* < .001) after the ACA and lowest for persons without disabilities living in a nonexpansion state before the ACA (74 191 of 426 387 [17.4%];* P* < .001). Private health insurance coverage was lower for persons with a disability by approximately 2-fold compared with persons without a disability, regardless of expansion state status. All of the associations reported in Table 2 were statistically significant.

**Table 2.** Weighted Percentage of Health Insurance Coverage by Self-reported Disability Status and State Medicaid Expansion to 2 549 376 Medicaid-Eligible Adults, 2010-2016^a^

Variable	Uninsured		Medicaid		Private Insurance		No.
	%	*P* Value	%	*P* Value	%	*P* Value	
**Pre-ACA, 2010-2013**							
Disability							
Expansion	20.4	<.001	61.1	<.001	15.2	<.001	228 036
No expansion	28.1	<.001	50.3	<.001	15.5	<.001	159 134
No disability							
Expansion	44.7	<.001	28.4	<.001	26.8	<.001	651 962
No expansion	55.5	<.001	17.4	<.001	26.3	<.001	426 387
**Post-ACA, 2014-2016**							
Disability							
Expansion	11.1	<.001	70.2	<.001	16.4	<.001	176 145
No expansion	23.1	<.001	52.7	<.001	18.7	<.001	119 609
No disability							
Expansion	27.0	<.001	43.0	<.001	30.5	<.001	477 822
No expansion	44.2	<.001	20.1	<.001	35.2	<.001	310 281

Abbreviation: ACA, Patient Protection and Affordable Care Act.

^a^ Medicaid-eligible defined as persons aged 26 to 64 years with income below 139% of the federal poverty level. Data are from the American Community Survey. All years are calendar years (January 1 through December 31).

Table 3, Table 4, and Table 5 provide the results of the triple-differences linear probability regression of insurance coverage for Medicaid-eligible respondents by disability status and residence in a Medicaid expansion state. The estimates in Table 3 indicate that Medicaid expansion was associated with a lower uninsured rate for persons both with a disability (7.1% − 16.2% = −9.1%) and without a disability (21.2% − 34.9% = −13.7%). However, the difference in the uninsurance rate was 2.0% greater for persons with a disability compared with persons without a disability (*P* < .001).

**Table 3.** Triple-Differences Model, Multivariate Linear Probability Estimates for No Health Insurance Coverage (Uninsured) by Self-reported Disability and State Medicaid Expansion Status Among 2 549 376 Medicaid-Eligible Adults, 2010-2016^a^

Variables	%
**Before Medicaid Expansion (2010-2013)**	
With disability	
Control group: no Medicaid expansion and disability^b^	25.1
Treatment group: Medicaid expansion and disability^b^	20.2
Treatment − control difference	−4.9
Without disability	
Control: no Medicaid expansion and no disability	49.9
Treatment: Medicaid expansion and no disability	42.4
Treatment − control difference	−7.5
With − without disability difference, 2010-2013^c^	2.6
**After Medicaid Expansion (2014-2016)**	
With disability	
Control: no Medicaid expansion and disability	16.2
Treatment: Medicaid expansion and disability	7.1
Treatment − control difference	−9.1
Without disability	
Control: no Medicaid expansion and no disability	34.9
Treatment: Medicaid expansion and no disability	21.2
Treatment − control difference	−13.7
With disability − without disability difference, 2014-2016^c^	4.6
**Triple Difference**	
After (2014-2016) − before (2010-2013) difference^c^	2.0
*R*^2^	16

^a^ Multivariate adjustment included the following control variables: age, sex, marital status, race/ethnicity, immigration status, poverty status, education, employment status, and metropolitan residence. Data are from the American Community Survey. All years are calendar years (January 1 through December 31). Medicaid-eligible defined as persons aged 26 to 64 years with income below 139% of the federal poverty level.

^b^ States that expanded Medicaid between January 1, 2014, and December 31, 2016, were classified as the treatment group, and states that did not expand Medicaid during the study period were classified as the control group.

^c^ *P* < .001

**Table 4.** Triple-Differences Model, Multivariate Linear Probability Estimates for Medicaid Coverage by Self-reported Disability and State Medicaid Expansion Status Among 2 549 376 Medicaid-Eligible Respondents, 2010-2016^a^

Variables	%
**Before Medicaid Expansion (2010-2013)**		
With disability	
Control group: no Medicaid expansion and disability^b^	42.9
Treatment group: Medicaid expansion and disability^b^	49.3
Treatment − control difference	6.4
Without disability	
Control: no Medicaid expansion and no disability	15.6
Treatment: Medicaid expansion and no disability	21.6
Treatment − control difference	6.0
With disability − without disability difference, 2010-2013^c^	0.4
**After Medicaid Expansion (2014-2016)**		
With disability	
Control: no Medicaid expansion and disability	49.3
Treatment: Medicaid expansion and disability	62.3
Treatment − control difference	13.0
Without disability	
Control: no Medicaid expansion and no disability	22.6
Treatment: Medicaid expansion and no disability	40.3
Treatment − control difference	17.7
With disability − without disability difference, 2014-2016^d^	−4.7
**Triple Difference**	
After (2014-2016) − before (2010-2013) difference^d^	−5.1
*R*^2^	18

^a^ Multivariate adjustment included the following control variables: age, sex, marital status, race/ethnicity, immigration status, poverty status, education, employment status, and metropolitan residence. Medicaid-eligible defined as persons aged 26 to 64 years with income below 139% of the federal poverty level. Data are from the American Community Survey. All years are calendar years (January 1 through December 31).

^b^ States that expanded Medicaid between January 1, 2014, and December 31, 2016, were classified as the treatment group, and states that did not expand Medicaid during the study period were classified as the control group.

^c^ *P* = .10.

^d^ *P* < .001.

**Table 5.** Triple-Differences Model, Multivariate Linear Probability Estimates for Private Health Insurance Coverage by Self-reported Disability and State Medicaid Expansion Status Among 2 549 376 Medicaid-Eligible Adults, 2010-2016^a^

Variables	%
**Before Medicaid Expansion (2010-2013)**	
With disability	
Control group: no Medicaid expansion and disability^b^	30.0
Treatment group: Medicaid expansion and disability^b^	33.2
Treatment − control difference	3.2
Without disability	
Control: no Medicaid expansion and no disability	35.5
Treatment: Medicaid expansion and no disability	39.6
Treatment − control difference	4.1
With disability − without disability difference, 2010-2013^c^	−0.9
**After Medicaid Expansion (2014-2016)**	
With disability	
Control: no Medicaid expansion and disability	33.4
Treated: Medicaid expansion and disability	34.6
Treatment − control difference	1.2
Without disability	
Control: no Medicaid expansion and no disability	44.2
Treated: Medicaid expansion and no disability	43.0
Treatment – control difference	−1.2
With disability − without disability difference, 2014-2016^c^	2.4
**Triple Difference**	
After (2014-2016) − before (2010-2013) difference^c^	3.3
*R*^2^	13

^a^ Multivariate adjustment included the following control variables: age, sex, marital status, race/ethnicity, immigration status, poverty status, education, employment status, and metropolitan residence. Medicaid-eligible defined as persons aged 26 to 64 years with income below 139% of the federal poverty level. Data are from the American Community Survey. All years are calendar years (January 1 through December 31).

^b^ States that expanded Medicaid between January 1, 2014, and December 31, 2016, were classified as the treatment group, and states that did not expand Medicaid during the study period were classified as the control group.

^c^ *P* < .001.

The estimates in Table 4 indicate that there was not a statistically significant difference in Medicaid coverage between persons with and without a disability before Medicaid expansion went into effect in 2014 (*P* = .10). However, there was a statistically significant difference in Medicaid coverage after 2014 between persons with and persons without a disability (13.0% − 17.7% = −4.7%; *P* < .001) associated with residence in a Medicaid expansion state. Although Medicaid expansion was associated with an increase in Medicaid coverage for both persons with a disability (49.3% before vs 62.3% after; change, 13.0%) and without a disability (21.6% before vs 40.3% after; change, 17.7%), the difference in reported Medicaid coverage was 5.1% lower for persons with a disability compared with persons without a disability (−4.7% minus 0.4%; *P* < .001).

The estimates in Table 5 indicate that there was a statistically significant difference in private insurance coverage rates between persons with and without a disability before Medicaid expansion went into effect in 2014. The difference in private coverage between persons living in a Medicaid expansion state before 2014 was less than 1 percentage point lower for persons with a disability compared with persons without a disability. However, after 2014, the association reversed as Medicaid expansion was associated with approximately a 2–percentage point increase in private coverage for persons with a disability compared with persons without a disability. Overall, Medicaid expansion was associated with a 3% higher private insurance rate for persons with a disability compared with persons without a disability.

The sensitivity tests suggest the triple-differences results are robust to model specification. We first reanalyzed the triple-differences models after dropping states that expanded Medicaid earlier than January 1, 2014, or later than December 31, 2014, and found that the estimates were similar suggesting that the results are robust to the timing of the Medicaid expansion. For the outcome of Medicaid coverage, we also determined that excluding persons eligible for Medicare and Medicaid (dual-eligible) did not substantively affect the results (−4.5% compared with −5.1%; *P* < .001 for both). Finally, we analyzed the triple-differences model for each type of self-reported disability. With Medicaid as the outcome variable, the triple difference estimate was −5% (*P* < .001) for persons with a cognitive disability, −5% (*P* < .001) for persons with an ambulatory disability, and −4% (*P* < .001) for persons with a sensory disability. With private insurance as the outcome variable, the triple difference estimate was 3% (*P* < .001) for persons with a cognitive disability, 3% (*P* < .001) for persons with an ambulatory disability, and 3% (*P* < .001) for persons with a sensory disability. The triple difference estimates for types of disability are similar to the estimates shown in Table 4 and Table 5, suggesting that the results are robust to the measurement of the outcome variable.

## Discussion

Earlier research has established that the ACA substantially decreased uninsurance rates in the United States, particularly for states that chose to expand Medicaid eligibility for their residents.^2,3^ Our hypothesis was confirmed. We found that persons with a disability living in Medicaid expansion states showed greater improvement in health insurance coverage compared with persons with a disability living in states that chose not to expand Medicaid.

However, the benefits of expansion have not been evenly distributed across demographic groups. Our analysis suggests that, although both groups benefited significantly from increasing Medicaid coverage in expansion states, persons without a disability had greater gains in Medicaid coverage than persons with a disability. One explanation for this finding may be that many persons with moderate to severe disability were already covered by Medicaid or Medicare before expansion of eligibility.^19,20,24,25,26^ For example, the average state poverty threshold for a person with disability to qualify for Medicaid was 85% of the federal poverty level compared with the higher threshold of 138% of the federal poverty level for persons without a disability.^16^ The improved eligibility criteria may not have been as much a driver of the lower uninsurance rate as “welcome mat” factors associated with the ACA, such as improved outreach efforts to sign up eligible individuals and financial incentives for individual and state participation.^34,35^ Our methodological approach contributes to this debate by isolating the specific contribution of Medicaid expansion on reducing the uninsurance rate for this population. There has been recent debate, with some Medicaid expansion opponents contending that expansion has led to a decrease in resources available for populations of persons with a disability and longer waiting lists for home and community-based care.^36,37^ However, Medicaid recipients without a disability are unlikely to be eligible for home care. More research is needed to explore this argument.

Medicaid expansion may have been associated with higher private insurance rates for persons with a disability relative to persons without a disability. Further research is needed to understand the reasons for this result. We speculate that the most likely cause of this association was the ACA’s provision to require coverage of preexisting conditions in private insurance plans. Another possibility is that persons with disabilities living in expansion states were more likely to be employed compared with the same population living in nonexpansion states.^16,20^ Possibly this association may have led more persons with a disability to find employment in jobs providing employer-sponsored health insurance in the expansion states, but this hypothesis requires further study.

### Limitations

There are several limitations to consider when interpreting the results of this study. First, disability has been measured in many ways across studies, and the ACS data do not use more clinically relevant measures such as Activities of Daily Living or Instrumental Activities of Daily Living.^24^ Furthermore, our measure is not directly comparable with the definition used by the Social Security Administration, because our measure of disability is self-reported rather than medically determined. Therefore, the results of this study should be interpreted cautiously until the findings are replicated using other measures of disability. Although we adjusted for a range of potential individual-level confounders that were available in the ACS, control variables may have been inadvertently omitted that could violate the parallel trends assumption. Related to this topic, there are many other policies or situations across states that could be associated with health insurance that were not measured in this study and were assumed to be held constant by our state and year fixed-effects triple-differences model analysis. We provided several sensitivity analyses in eTables 2 through 6 in the Supplement to demonstrate that the results are robust to different analytic approaches and assumptions.

## Conclusions

The findings of our study suggest that Medicaid expansion was associated with lower uninsurance rates and greater rates of Medicaid and private insurance coverage for persons with a disability. Despite these gains in health insurance coverage for persons with a disability, we found gaps in insurance coverage for this population that persisted despite improved coverage eligibility in expansion states. Persons with a disability have greater health care needs and limited resources to access care^24,30^; thus, additional policies and resources should be identified to mitigate risks of uninsurance for this population in expansion states.^25,26,38,39,40,41^

## Notes

Supplement. **eTable 1.** Weighted Characteristics of Medicaid-Eligible Respondents, American Community Survey 2010–16 (N=2,549,376) **eFigure.** Weighted Trend in Insurance Coverage for Medicaid-Eligible Adults (Age 26-64 and Income Below 139% of the Federal Poverty Level) by Type of Disability, American Community Survey 2010–16 (N=2,549,376) **eAppendix.** Parallel Trends Test **eTable 2.** Sensitivity Analysis: Excluding Early and Late Medicaid Expansion States **eTable 3.** Sensitivity Analysis: Excluding Early and Late Medicaid Expansion States **eTable 4.** Sensitivity Analysis: Excluding Early and Late Medicaid Expansion States **eTable 5.** Sensitivity Analysis: Types of Disability − Medicaid Coverage **eTable 6.** Sensitivity Analysis: Types of Disability − Private Health Insurance
