# Supplementary material for: Association of Medicaid Expansion With Health Insurance Coverage Among Persons With a Disability
Source: JAMA Netw Open. 2019 Jul 17;2(7):e197136. doi: 10.1001/jamanetworkopen.2019.7136 (PMC6647921; doi:10.1001/jamanetworkopen.2019.7136)
Supplement: Supplement. — eTable 1. Weighted Characteristics of Medicaid-Eligible Respondents, American Community Survey 2010–16 (N=2,549,376) eFigure. Weighted Trend in Insurance Coverage for Medicaid-Eligible Adults (Age 26-64 and Income Below 139% of the Federal Poverty Level) by Type of Disability, American Community Survey 2010–16 (N=2,549,376) eAppendix. Parallel Trends Test eTable 2. Sensitivity Analysis: Excluding Early and Late Medicaid Expansion States eTable 3. Sensitivity Analysis: Excluding Early and Late Medicaid Expansion States eTable 4. Sensitivity Analysis: Excluding Early and Late Medicaid Expansion States eTable 5. Sensitivity Analysis: Types of Disability − Medicaid Coverage eTable 6. Sensitivity Analysis: Types of Disability − Private Health Insurance [file jamanetwopen-2-e197136-s001.pdf]

Supplementary Online Content

Stimpson JP, Kemmick Pintor J, McKenna RM, Park S, Wilson FA. Association of Medicaid expansion with health insurance coverage among persons with a disability. *JAMA Netw Open.* 2019;2(7):e197136. doi:10.1001/jamanetworkopen.2019.7136

eTable 1. Weighted Characteristics of Medicaid-Eligible Respondents, American Community Survey 2010–16 (N=2,549,376)

eFigure. Weighted Trend in Insurance Coverage for Medicaid-Eligible Adults (Age 26-64 and Income Below 139% of the Federal Poverty Level) by Type of Disability, American Community Survey 2010–16 (N=2,549,376)

eAppendix. Parallel Trends Test

eTable 2. Sensitivity Analysis: Excluding Early and Late Medicaid Expansion States

eTable 3. Sensitivity Analysis: Excluding Early and Late Medicaid Expansion States

eTable 4. Sensitivity Analysis: Excluding Early and Late Medicaid Expansion States

eTable 5. Sensitivity Analysis: Types of Disability − Medicaid Coverage

eTable 6. Sensitivity Analysis: Types of Disability − Private Health Insurance

This supplementary material has been provided by the authors to give readers additional information about their work.

© 2019 Stimpson JP et al. *JAMA Network Open*.

© 2019 Stimpson JP et al. *JAMA Network Open*.

eTable 1. Weighted characteristics of Medicaid-eligible respondents, American Community Survey 2010–16 (N=2,549,376)

	%	Lower 95% CI	Upper 95% CI
Uninsured	37.14%	37.07%	37.22%
Medicaid	35.77%	35.70%	35.85%
Private Insurance	25.97%	25.91%	26.04%
Number of Disabilities			
0	76.00%	75.94%	76.07%
1	8.11%	8.06%	8.15%
2 or more	15.89%	15.84%	15.95%
Female	52.90%	52.82%	52.98%
Age Groups			
26-44	56.86%	56.78%	56.94%
45-64	43.14%	43.06%	43.22%
Income < 100% FPL	73.50%	73.43%	73.57%
Married	30.89%	30.82%	30.97%
Race/Ethnicity			
Non-Hispanic White	47.85%	47.77%	47.93%
Hispanic	24.35%	24.28%	24.42%
Black	19.55%	19.48%	19.61%
Other Race/Ethnicity	8.26%	8.22%	8.30%
Immigration Status			
US Native	76.21%	76.14%	76.28%
Naturalized Citizen	7.18%	7.14%	7.22%
Non-Citizen	16.61%	16.54%	16.67%
Education			
Less than High School	23.66%	23.59%	23.73%
High School	43.75%	43.68%	43.83%
Some College	21.28%	21.22%	21.35%
College	11.30%	11.25%	11.35%
Not Employed	61.16%	61.08%	61.23%
Metropolitan	76.01%	75.94%	76.07%

© 2019 Stimpson JP et al. *JAMA Network Open*.

eFigure. Weighted Trend in Insurance Coverage for Medicaid-eligible adults (age 26-64 and income below 139% of the federal poverty level) by Type of Disability, American Community Survey 2010–16 (N=2,549,376)

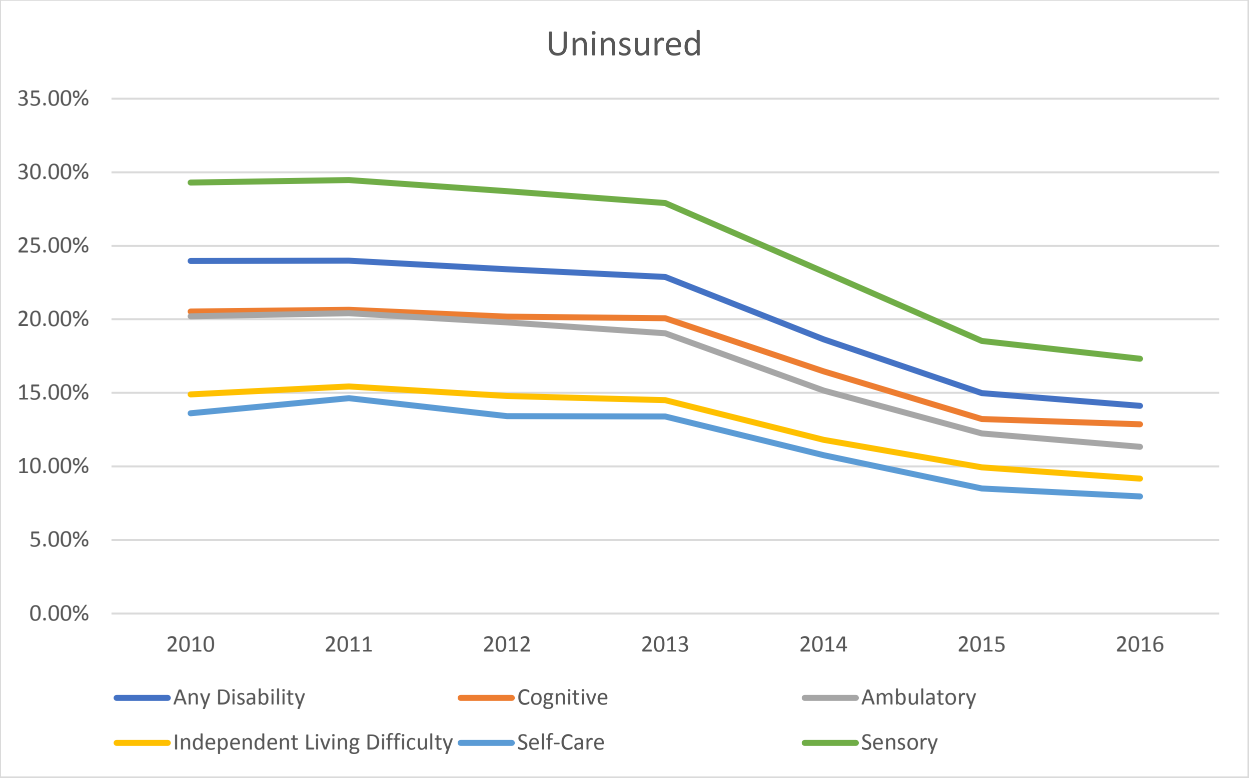

© 2019 Stimpson JP et al. *JAMA Network Open*.

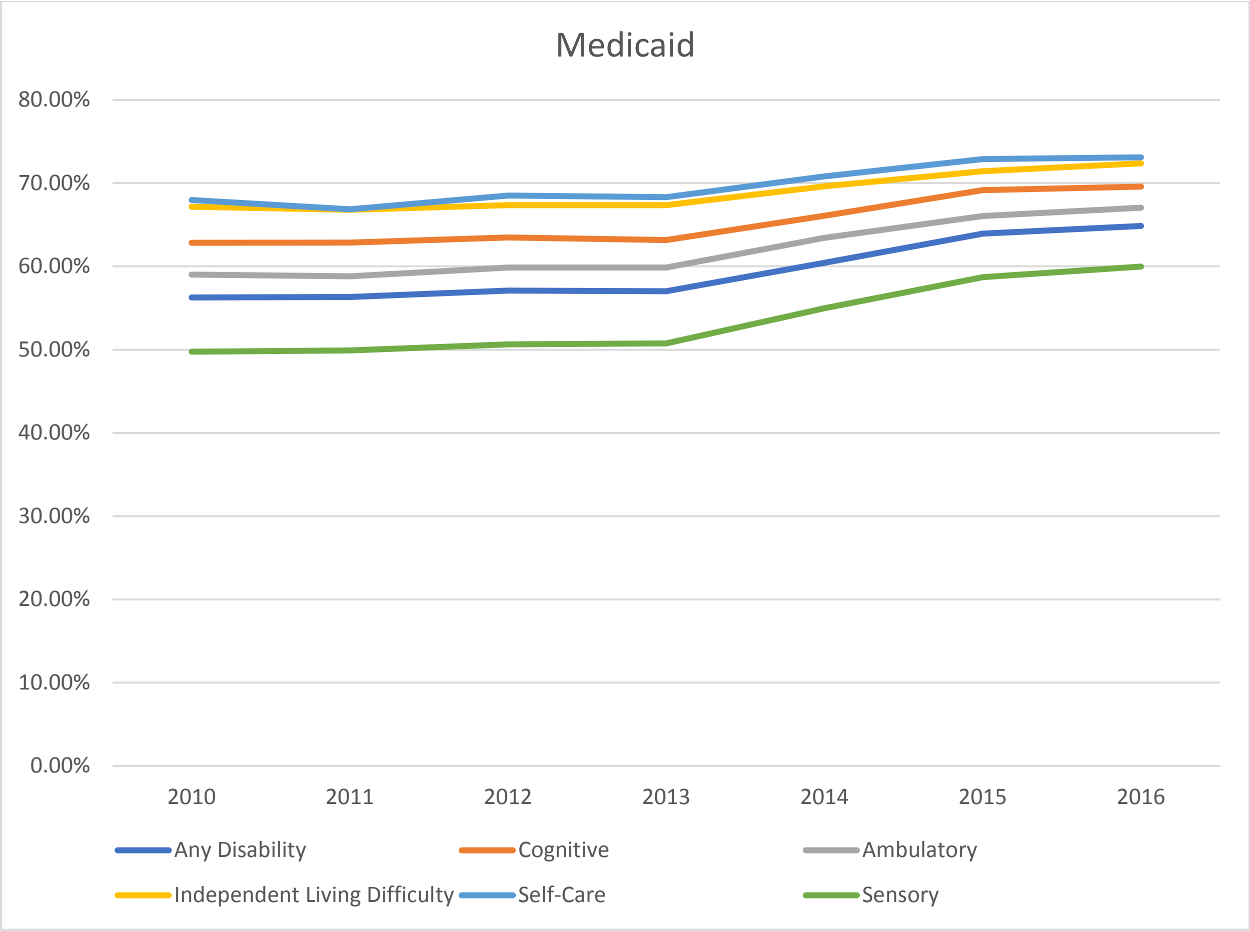

© 2019 Stimpson JP et al. *JAMA Network Open*.

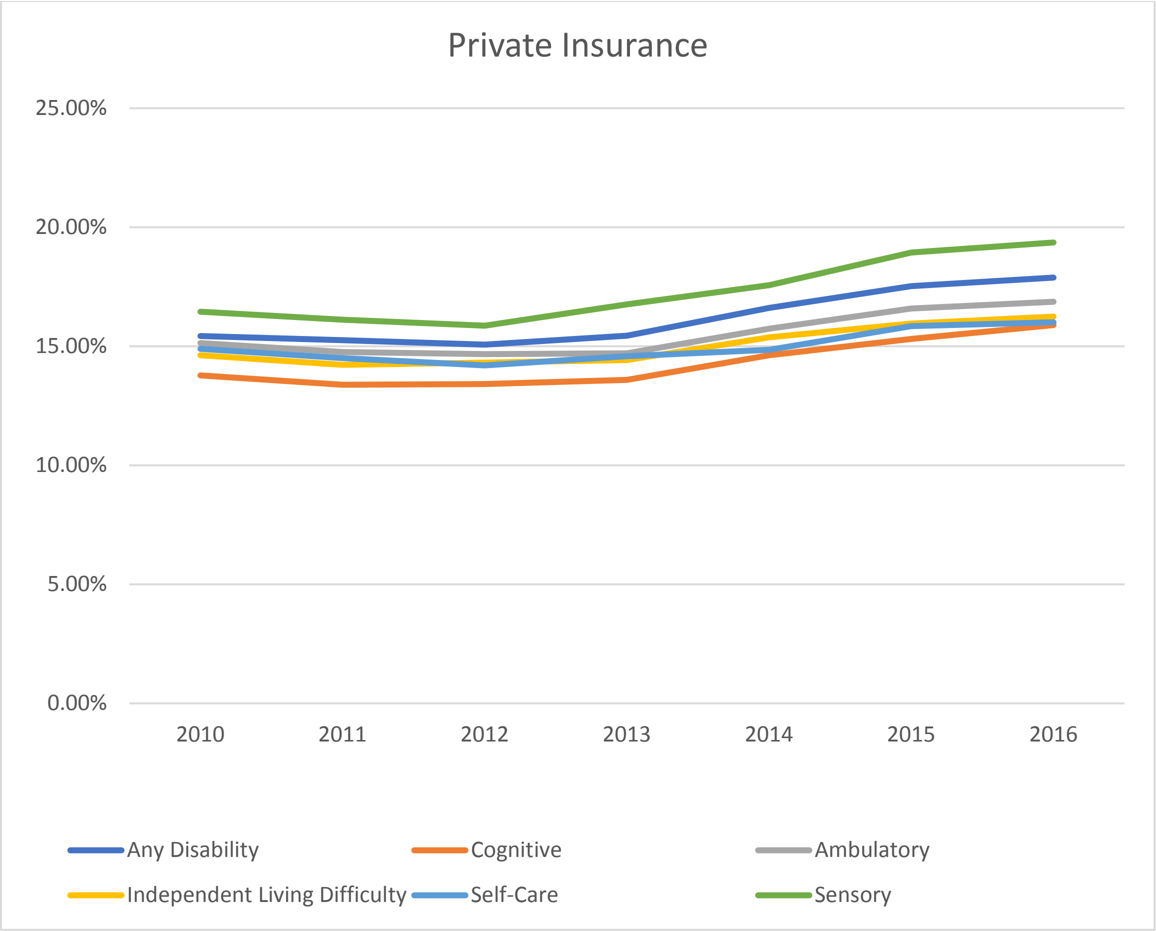

© 2019 Stimpson JP et al. *JAMA Network Open*.

eAppendix. Parallel Trends Test

First, we provided graphic evidence using weighted trends in uninsured for expansion and non-expansion states by disability status. Second, we calculated the coefficients on the triple interaction and then calculated a joint F-test across the coefficients for the pre-expansion period (2010-2013). The following is a summary of the chi square tests:

Year x Expansion State x Disability Status: chi2 = 46.02

Year x Disability Status: chi2 = 25.64

Year x Medicaid Expansion: chi2 = 1.90

Weighted Trend in Percent Uninsured Stratified by Residence in Medicaid or Non-Medicaid Expansion States and Disability Status: American Community Survey, 2010-2016

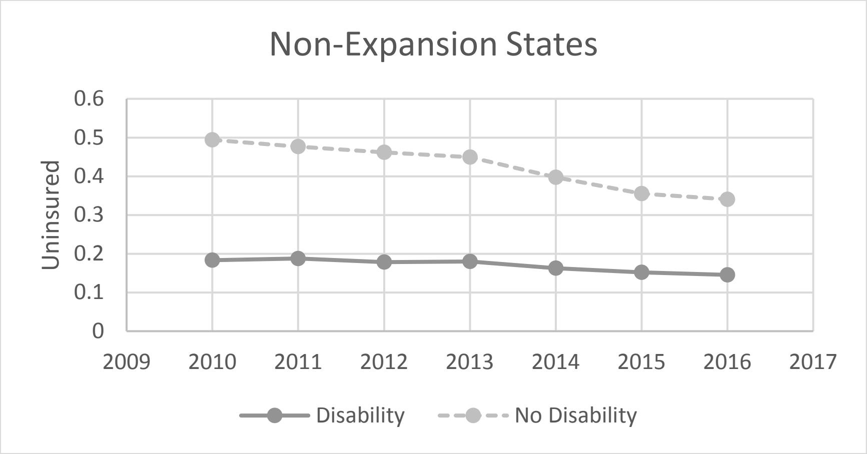

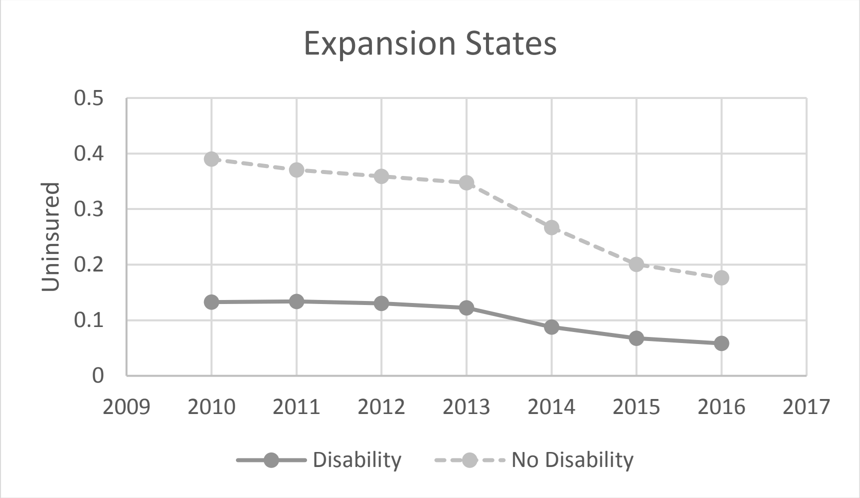

© 2019 Stimpson JP et al. *JAMA Network Open*.

eTable 2. Sensitivity Analysis: Excluding Early and Late Medicaid Expansion States

Triple differences estimates for no health insurance coverage by self-reported disability and state Medicaid expansion status, American Community Survey 2010-16, N = 2,549,376 Medicaid eligible respondents

	Coeff.	SE	t	P>|t|
Before				
Control - No Medicaid Expansion & Disability	0.25			
Control - No Medicaid Expansion & No Disability	0.498			
Treated - Medicaid Expansion & Disability	0.207			
Treated - Medicaid Expansion & No Disability	0.419			
Difference (Treatment-Control)	0.035	0.002	14.12	0.000
After				
Control - No Medicaid Expansion & Disability	0.164			
Control - No Medicaid Expansion & No Disability	0.352			
Treated - Medicaid Expansion & Disability	0.078			
Treated - Medicaid Expansion & No Disability	0.217			
Difference (Treatment-Control)	0.048	0.003	18.87	0.000
DDD	0.013	0.004	3.68	0.000
Covariates				
Male	0.098	0.001	113.476	0.000
Age 45-64	-0.037	0.001	-41.298	0.000
In poverty	0.066	0.001	65.587	0.000
Married	-0.07	0.001	-72.182	0.000
Hispanic	0.057	0.002	37.612	0.000
Black	-0.029	0.001	-24.362	0.000
Other race/ethnicity	-0.02	0.002	-10.475	0.000
Naturalized Citizen	0.022	0.002	10.727	0.000
Non-Citizen	0.243	0.002	145.009	0.000
High School	-0.02	0.001	-17.563	0.000
Some college	-0.052	0.001	-37.688	0.000
College	-0.115	0.002	-70.268	0.000
Unemployed	0.011	0.001	11.15	0.000
Metropolitan	-0.022	0.001	-20.976	0.000

Linear probability model estimates are based on the sample weights provided by the Census Bureau and adjusted for state and year fixed effects. Medicaid eligibility was defined by age 26-64 and income below 139% of the federal poverty level. Multivariate adjustment included the following control variables: age, sex, marital status, race/ethnicity, immigration status, poverty status, education, employment status, and metropolitan residence.

© 2019 Stimpson JP et al. *JAMA Network Open*.

eTable 3. Sensitivity Analysis: Excluding Early and Late Medicaid Expansion States

Triple differences estimates for Medicaid coverage by self-reported disability and state Medicaid expansion status, American Community Survey 2010-16, N = 2,549,376 Medicaid eligible respondents

	Coeff.	S. Err.	t	P>|t|
Before				
Control - No Medicaid Expansion & Disability	0.43			
Control - No Medicaid Expansion & No Disability	0.158			
Treated - Medicaid Expansion & Disability	0.477			
Treated - Medicaid Expansion & No Disability	0.22			
Difference (Treatment-Control)	-0.016	0.003	5.92	0.000
After				
Control - No Medicaid Expansion & Disability	0.489			
Control - No Medicaid Expansion & No Disability	0.222			
Treated - Medicaid Expansion & Disability	0.606			
Treated - Medicaid Expansion & No Disability	0.4			
Difference (Treatment-Control)	-0.061	0.003	20.16	0.000
DDD	-0.045	0.004	-11.38	0.000
Covariates				
Male	-0.094	0.001	-115.539	0.000
Age 45-64	-0.042	0.001	-50.035	0.000
In poverty	0.063	0.001	67.662	0.000
Married	0.003	0.001	3.808	0.000
Hispanic	0.006	0.001	4.003	0.000
Black	0.063	0.001	55.265	0.000
Other race/ethnicity	0.035	0.002	19.801	0.000
Naturalized Citizen	-0.009	0.002	-4.461	0.000
Non-Citizen	-0.15	0.001	-103.812	0.000
High School	-0.053	0.001	-47.711	0.000
Some college	-0.082	0.001	-62.516	0.000
College	-0.17	0.001	-115.631	0.000
Unemployed	0.097	0.001	106.196	0.000
Metropolitan	-0.008	0.001	-7.67	0.000

Linear probability model estimates are based on the sample weights provided by the Census Bureau and adjusted for state and year fixed effects. Medicaid eligibility was defined by age 26-64 and income below 139% of the federal poverty level. Multivariate adjustment included the following control variables: age, sex, marital status, race/ethnicity, immigration status, poverty status, education, employment status, and metropolitan residence. Persons dual-eligible for Medicare are excluded.

© 2019 Stimpson JP et al. *JAMA Network Open*.

eTable 4. Sensitivity Analysis: Excluding Early and Late Medicaid Expansion States

Triple differences estimates for private health insurance coverage and employer/union based coverage by self-reported disability and state Medicaid expansion status, American Community Survey 2010-16, N = 2,549,376 Medicaid eligible respondents

	**Private**			
	Coeff.	S. Err.	t	P>|t|
Before				
Control - No Medicaid Expansion & Disability	0.304			
Control - No Medicaid Expansion & No Disability	0.357			
Treated - Medicaid Expansion & Disability	0.342			
Treated - Medicaid Expansion & No Disability	0.401			
Difference (Treatment-Control)	-0.006	0.002	3.01	0.003
After				
Control - No Medicaid Expansion & Disability	0.339			
Control - No Medicaid Expansion & No Disability	0.446			
Treated - Medicaid Expansion & Disability	0.356			
Treated - Medicaid Expansion & No Disability	0.431			
Difference (Treatment-Control)	0.031	0.003	12.1	0.000
DDD	0.038	0.003	11.31	0.000
Covariates				
Male	-0.029	0.001	-36.305	0.000
Age 45-64	0.037	0.001	44.911	0.000
In poverty	-0.097	0.001	-95.819	0.000
Married	0.083	0.001	89.215	0.000
Hispanic	-0.061	0.001	-44.623	0.000
Black	-0.029	0.001	-27.292	0.000
Other race/ethnicity	-0.01	0.002	-5.627	0.000
Naturalized Citizen	-0.012	0.002	-5.977	0.000
Non-Citizen	-0.102	0.001	-68.955	0.000
High School	0.066	0.001	69.937	0.000
Some college	0.116	0.001	95.775	0.000
College	0.278	0.002	172.829	0.000
Unemployed	-0.148	0.001	-157.251	0.000
Metropolitan	0.032	0.001	34.111	0.000

© 2019 Stimpson JP et al. *JAMA Network Open*.

	Employer			
	Coeff.	S. Err.	t	P>|t|
Before				
Control - No Medicaid Expansion & Disability	0.268			
Control - No Medicaid Expansion & No Disability	0.307			
Treated - Medicaid Expansion & Disability	0.269			
Treated - Medicaid Expansion & No Disability	0.31			
Difference (Treatment-Control)	-0.002	0.002	1.34	0.18
After				
Control - No Medicaid Expansion & Disability	0.271			
Control - No Medicaid Expansion & No Disability	0.336			
Treated - Medicaid Expansion & Disability	0.267			
Treated - Medicaid Expansion & No Disability	0.318			
Difference (Treatment-Control)	0.014	0.002	6.65	0.000
DDD	0.016	0.003	5.96	0.000
Covariates				
Male	-0.029	0.001	-41.567	0.000
Age 45-64	0.007	0.001	9.806	0.000
In poverty	-0.084	0.001	-90.096	0.000
Married	0.07	0.001	82.726	0.000
Hispanic	-0.018	0.001	-14.757	0.000
Black	0.01	0.001	10.263	0.000
Other race/ethnicity	-0.002	0.002	-1.326	0.185
Naturalized Citizen	-0.021	0.002	-11.69	0.000
Non-Citizen	-0.094	0.001	-72.062	0.000
High School	0.045	0.001	54.867	0.000
Some college	0.067	0.001	63.44	0.000
College	0.136	0.001	95.67	0.000
Unemployed	-0.157	0.001	-184.895	0.000
Metropolitan	0.017	0.001	20.69	0.000

Linear probability model estimates are based on the sample weights provided by the Census Bureau and adjusted for state and year fixed effects. Medicaid eligibility was defined by age 26-64 and income below 139% of the federal poverty level. Multivariate adjustment included the following control variables: age, sex, marital status, race/ethnicity, immigration status, poverty status, education, employment status, and metropolitan residence.

© 2019 Stimpson JP et al. *JAMA Network Open*.

eTable 5. Sensitivity Analysis: Types of Disability – Medicaid Coverage

Triple differences estimates for Medicaid coverage by self-reported disability and state Medicaid expansion status, American Community Survey 2010-16, N = 2,549,376 Medicaid eligible respondents

	**Cognitive**			**Ambulatory**			**Sensory**		
	Coeff.	SE	t	Coeff.	SE	t	Coeff.	SE	t
Before									
Difference (Treatment - Control)	0.000	0.003	0.04	-0.002	0.003	0.61	0.012	0.004	3.17
After									
Difference (Treatment - Control)	-0.047	0.003	13.63	-0.052	0.003	15.67	-0.027	0.004	6.14
DDD	-0.047	0.005	-10.22	-0.05	0.004	-11.44	-0.04	0.006	-6.72

© 2019 Stimpson JP et al. *JAMA Network Open*.

eTable 6. Sensitivity Analysis: Types of Disability – Private Health Insurance

Triple differences estimates for private health insurance coverage by self-reported disability and state Medicaid expansion status, American Community Survey 2010-16, N = 2,549,376 Medicaid eligible respondents

	**Cognitive**			**Ambulatory**			**Sensory**		
	Coeff.	SE	t	Coeff.	SE	t	Coeff.	SE	t
Before									
Difference (Treatment - Control)	-0.011	0.002	5.04	-0.006	0.002	2.94	-0.008	0.003	2.65
After									
Difference (Treatment - Control)	0.019	0.003	6.89	0.023	0.003	8.52	0.024	0.004	6.68
DDD	0.03	0.004	8.54	0.029	0.003	8.47	0.032	0.005	6.83

© 2019 Stimpson JP et al. *JAMA Network Open*.
